# Editorial Department

**Published:** 1874-12-15

**Authors:** 


### Editorial Department.

THE present number completes the fifteenth volume of the MEDICAL EXAMINER.

It was commenced by the present senior editor in January, 1860; and, with the exception of a month immediately following the great fire of Oct., 1871, when a printing press was not left in Chicago capable of doing the work, it has visited its patrons with regularity and reasonable promptness. It has always been the personal property of its editors, owing allegiance to no other interest or institution, and having for its great leading object the promotion of the educational and scientific interests of the profession, and direct practical aid to the practitioner at the bedside of his patient. In furtherance of the last-named object the senior editor has ever contributed freely (perhaps some would say too freely) such clinical facts and observations concerning the causes, nature, and treatment, of all the more important diseases coming before him in the ample field of both private and hospital practice, as he deemed most valuable to its readers. With these same objects steadily in view, we propose, as stated in the last preceding number, to give our readers in the volume for 1875, one hundred pages more of reading matter, presented in as good style as any medical periodical in this country.

We approach the beginning of the new year with all our arrangements for giving our readers a valuable medical periodical more complete than ever before.

Let none forget that *now* is the time for renewing their subscriptions, and speaking a good word for the EXAMINER to their neighbors in the profession.

---

DISSECTING MATERIAL. — During the last two months quite a number of applications have come to us and to the demonstrators of anatomy in the medical colleges, asking to have *subjects* sent to them for dissection and anatomical study. These applications are prompted by the supposition that the law passed by the last legislature, legalizing the study of anatomy, would place more *subjects* at the disposal of the teachers of anatomy here than were actually needed for their own use. This is a mistake.

On careful inquiry we find the practical operation of the law, thus far, has not supplied a single subject more than was actually needed by the classes in the several medical colleges in this city. Besides, the law expressly prohibits all *traffic* in such *material*, and the faculties of our colleges are honorably pledged to the public authorities to abide strictly by the terms of the law. We would like to have all our friends in other places supplied, but cannot aid them at present.

---

INDEX to Volume XV. with January 1st number.

